# Nestin Expression and the Survival of Patients With Digestive Tract Cancers: A Systematic Review and Meta-Analysis

**DOI:** 10.5152/tjg.2023.22485

**Published:** 2023-09-01

**Authors:** Lumi Huang, Lihui Chen, Yiming Wang, Nan Shan, Ting Wang, Dairong Li, Chunmei Wang, Huiwen Ma

**Affiliations:** 1 Department of Oncology, Chongqing University Cancer Hospital, Chongqing, China; 2 Department of Gastroenterological Surgery, Chongqing University Cancer Hospital, Chongqing, China; 3 Department of Gynaecology and Obstetrics, The First Affiliated Hospital of Chongqing Medical University, Chongqing, China

**Keywords:** Digestive tract cancer, nestin, immunohistochemistry, survival, meta-analysis

## Abstract

**Background/Aims:**

Several cancers have been associated with poor prognoses based on nestin, a confirmed marker of cancer stem cells. However, there is conflicting evidence regarding the prognostic value of tumor nestin expression in patients with digestive tract cancers. An investigation of the association between nestin and survival in patients with digestive tract cancers was performed in this meta-analysis.

**Materials and Methods:**

Meta-analyses were conducted using PubMed, Embase, and Web of Science databases to search for cohort studies. We analyzed the data using a random-effects model that incorporates differences between studies.

**Results:**

The pooled analysis showed a negative association between nestin expression and overall survival (hazard ratio: 1.38, 95% CI: 1.11 to 1.72, *P* = .004, I2 = 68%) and disease-free survival (hazard ratio: 1.48, 95% CI: 1.12 to 1.96, *P* = .005, I2 = 56%). Subgroup analysis showed that nestin expression was associated with poorer overall survival in gastric cancer (hazard ratio: 1.46, *P* < .001) and liver cancer (hazard ratio: 2.05, *P* < .001) patients, but not in colorectal cancer (hazard ratio: 1.03, *P* = .89) or pancreatic cancer (hazard ratio: 0.96, *P* = .80) patients. Further subgroup analysis showed a consistent association between nestin expression and poor overall survival in Asian and non-Asian studies, and in studies with univariate and multivariate regression models.

**Conclusion:**

To sum up, the presence of high nestin expression in digestive tract cancer patients is associated with poorer survival, particularly in patients with gastric and liver cancers.

**Main Points**

- The association between tumor nestin expression and survival of digestive tract cancer patients was evaluated by meta-analysis.
- Overall, high nestin expression was associated with poor overall and disease-free survival of these patients.
- Among subgroup analyses, gastric and liver cancers were primarily responsible for the association, but colorectal and pancreatic cancers were not.

## INTRODUCTION

Digestive tract cancers (DTCs) are a group of heterogeneous cancers that mainly include esophagus cancer (EC), gastric cancer (GC), colorectal cancer (CRC), as well as hepatobiliary and pancreatic cancers (PCs).^1,2^ It has been shown that DTCs remain the important cause of cancer-related death of the global population.^3-6^ Current treatments for DTCs are comprehensive and complicated, preferably with surgical resection and combined with other strategies such as chemotherapy, radiotherapy, targeted therapy, and immunotherapy.^7-13^ However, the prognoses for patients with DTCs are still poor, particularly for those in the developing countries.^14^ Accordingly, the identification of prognostic markers for patients with DTCs is of great clinical importance.

Nestin is a class VI intermediate filament protein which could be detected in neural progenitor cells within the process of embryonic development and in various cancer tissues, including DTCs.^15,16^ In the human genome, nestin is found on chromosome 1’s long arm (q).^17^ The nestin gene consists of 4 exons separated by 3 introns. There are 1621 amino acids in the nestin protein, which has a predicted molecular weight of 177.4 kDa and a similar structure to other intermediate filament proteins.^17^ Previous studies showed that nestin is a potential marker for cancer stem cells (CSCs) and a useful marker of microvessel density in malignancies.^18-20^ Accumulating evidence indicates that higher expression of nestin in cancer tissue is related to poor survival in patients with certain types of cancers, such as ovarian cancer,^21^ nonsmall cell lung cancer (NSCLC),^22^ breast cancer,^23^ and glioma.^24^ There is, however, controversy over the association between nestin expression and survival outcomes in patients with DTCs,^25-38^ and the potential prognostic role of nestin in DTCs has not been comprehensively evaluated in previous meta-analyses. Thus, a systematic review and meta-analysis of patients with DTCs was conducted in order to comprehensively evaluate the possible link between nestin expression and survival. Additionally, subgroup analyses examined the influences of cancer types and ethnicity of the patients.

## MATERIAL AND METHODS

The meta-analysis was conceptualized, conducted, and reported in accordance with the Cochrane Handbook^39^ and MOOSE (Meta-analysis of Observational Studies in Epidemiology)^40^ MOOSE guidelines. The protocol of the meta-analysis has been registered at INPLASY (International Platform of Registered Systematic Review and Meta-analysis Protocols, https://inplasy.com/) with the registration number INPLASY202280087.

### Literature Search and Review

We conducted a systematic search of electronic databases, including PubMed, Web of Science, and Embase, from their inception to March 28, 2022, using the combined search terms as below: (1) “nestin,” (2) “esophagus” OR “esophageal” OR “esophagus” OR “gullet” OR “colon” OR “colorectal” OR “rectal” OR “anal” OR “pancreas” OR “pancreatic” OR “liver” OR “hepatic” OR “biliary duct” OR “bile duct” OR “gastric” OR “stomach” OR “cardia” OR “digestive tract,” and (3) “cancer” OR “carcinoma” OR “adenoma” OR “adenocarcinoma” OR “malignancy” OR “tumor” OR “tumour” OR “neoplasm.” Eligible studies had to have human subjects and be published in English as full-length papers. A manual screening of citation lists of related reviews and articles was also performed as a supplement to avoid missing any relevant studies. Two researchers independently conducted the literature search.

### Study Inclusion and Exclusion Criteria

To be included in this meta-analysis, studies must meet all the following criteria: (1) cohort studies; (2) patients with a confirmed diagnosis of DTCs were enrolled; (3) tumor expression of nestin was detected for each patient, via immunohistochemistry (IHC) or quantitative real-time polymerase chain reaction; (4) potential associations between nestin and overall survival (OS) and/or disease-free survival (DFS) were investigated; and (5) hazard ratio (HR) for the association was reported. Cutoffs for defining higher nestin expression (nestin positive) and lower nestin expression (nestin negative) were consistent with those used in the original studies. Exclusion criteria included reviews, editorials, preclinical studies, studies not in patients with DTCs, studies not evaluating nestin expression, or studies without survival outcomes.

### Data Collection and Study Quality Assessment

Data collection and quality assessment were also independently conducted by 2 researchers for each study included according to predetermined criteria. The corresponding author should be consulted if disagreements between the 2 researchers exist. We extracted data regarding study characteristics and outcomes using a predesigned Excel dataset form. Specifically, the following data were extracted and collected: (1) name of the first author, publication year, and country of the study; (2) study design (retrospective or prospective); (3) characteristics of the patients, including types of DTCs, sample size, number of patients with higher nestin expression, age, and sex; (4) methods for the detection of nestin expression in DTCs tissue and cutoffs for the definition of higher versus lower expression of nestin; (5) survival outcomes reported; and (6) adjustment of confounding factors in multivariate regression models. The Newcastle–Ottawa Scale (NOS)^41^ was used for the assessment of the study quality. Among the 3 domains of the scale, study group selection, comparison between groups, and outcome assessment, it rates studies on a scale of 1 to 9. The higher the NOS score, the better the study was.

### Statistical Analysis

The prognostic efficacy of nestin expression in cancer for the survival of patients with DTCs was presented as HRs and 95% CIs. From the original studies, HRs and corresponding (SEs) were extracted or calculated on the basis of 95% CIs or *P* values for subsequent analyses. Then, HRs and SEs were logarithmically transformed in order to normalize the distribution and stabilize the variance of the data.^39^ The between-study heterogeneity was assessed by the Cochrane’s *Q* test, and the estimated *I* ^2^ statistic.^42^ An *I* ^2^ >50% reflects a significant heterogeneity. A random-effect model was chosen for the synthesis of the HRs because this model has incorporated the possible influence of between-study heterogeneity, which therefore could retrieve a more generalized result.^39^ Sensitivity analyses omitting 1 individual study at a time were also performed to test the robustness of the findings.^43^ Subgroup analyses according to the types of DTCs, patient ethnicity, and analytic models (univariate or multivariate) were also conducted. Funnel plots were constructed for the analyses of possible publication bias. The risk of publication bias was firstly estimated by visual assessment of the symmetry of the funnel plots, and next evaluated with the Egger’s regression asymmetry test.^44^ *P* <.05 indicates statistical significance. RevMan (Version 5.1; Cochrane Collaboration, Oxford, UK) and Stata software were used for the statistical analyses.

## RESULTS

### Literature Search

**Figure 1.**
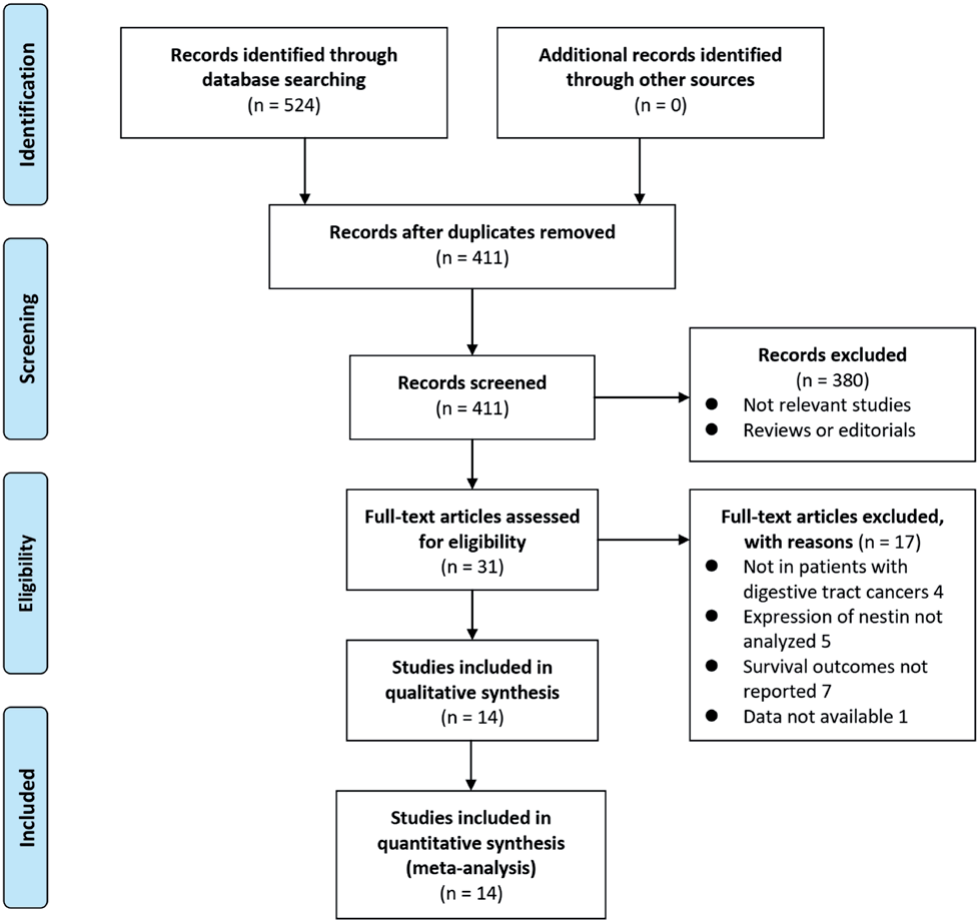
Flowchart of literature search.

According to Figure 1, the process of database search can be summarized as follows. In the initial literature search, 524 articles were found in PubMed, Embase, and Web of Science, and 411 were identified after duplicate records were excluded. A screening of the titles and abstracts excluded 380 articles due to their nonrelevance to the meta-analysis. Afterward, 31 potential relevant records underwent full-text reviews. Of these, 17 were further excluded due to the reasons listed in Figure 1. Finally, 14 studies were analyzed subsequently.^25-38^

### Study Characteristics and Quality Evaluation

A summary of the characteristics of the included studies can be found in Table 1. The included studies were all retrospective cohort studies published between 2002 and 2021 and carried out in China,^28,32-36^ Japan,^26,27^ Korea,^25,30^ Czech,^29^ Poland,^37^ Germany,^31^ and Switzerland.^38^ As for the types of DTCs, patients with GC,^25,33,36^ CRC,^26,37,38^ liver cancer (LC),^28,31,35^ PC,^27,29,30^ esophageal cancer,^32^ and ampullary cancer^34^ were included. There were 492 to 592 participants in the studies were included. Overall, 2453 patients with DTCs were included in this meta-analysis, and 884 (36%) of them were with higher expression of nestin in cancer tissue. For all of the included studies, IHC was applied to detect the expression of nestin in cancer tissue, and various cutoffs were applied to define the higher expression of nestin, such as above medians, or nestin expression in >10%, 20%, or 50% of the tumor cells. The follow-up durations varied between 20 and 84 months. The outcome of OS was reported in 11 studies,^25,27-31,33,35-38^ while the outcome of DFS was reported in 6 studies.^26,28,32,34,35,37^ In 6 studies, the association between nestin expression and survival of DTCs was estimated with univariate analyses,^26,27,29,31,33,37^ while in the other 8 studies,^25,28,30,32,34-36,38^ multivariate analyses were applied. Various confounding factors were adjusted to varying degrees in studies with multivariate analyses, including age, gender, cancer stage, histological grade, metastasis status, and adjuvant treatments. Studies included in the analysis averaged 6-8 NOS scores, indicating generally good quality studies (Table 2).

### Nestin Expression and Survival of Patients With Digestive Tract Cancers

**Figure 2.**
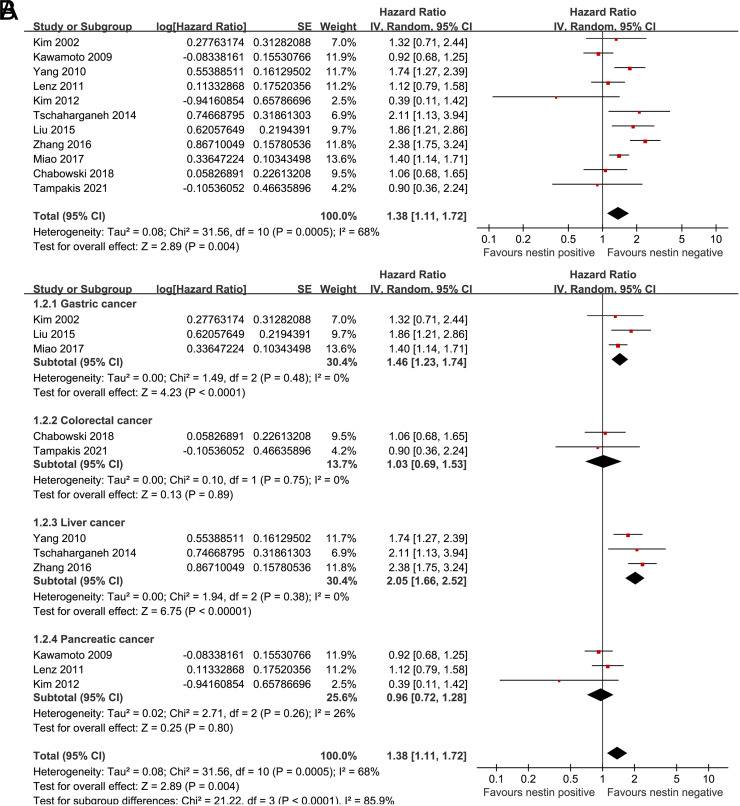
Forest plots for the meta-analysis of the association between nestin expression and OS in patients with DTCs. (A) Overall meta-analysis and (B) subgroup analysis according to the type of DTCs. DTCs, digestive tract cancers; OS, overall survival.

**Figure 3.**
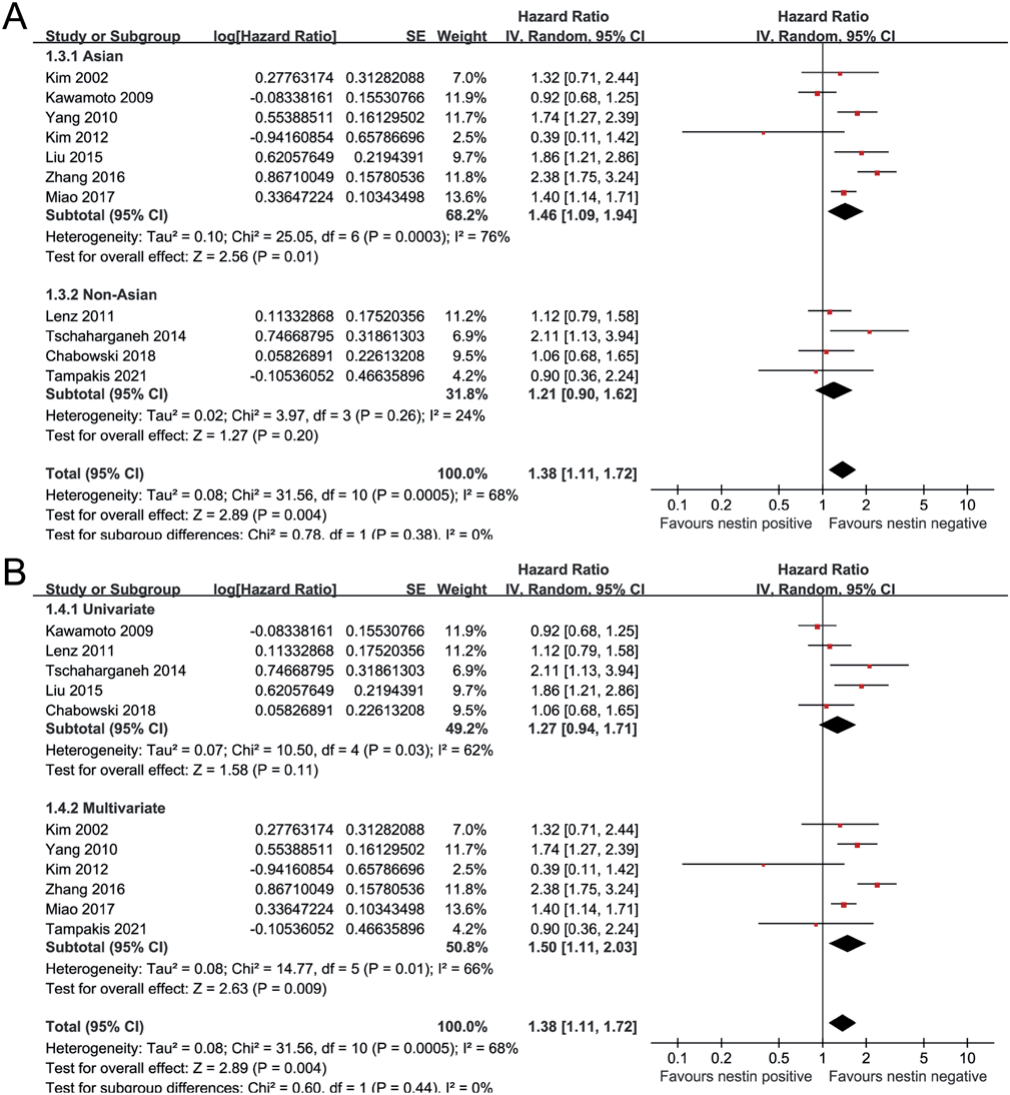
Forest plots for the subgroup analysis of the association between nestin expression and OS in patients with DTCs. (A) Subgroup analysis according to the ethnicity and (B) subgroup analysis in studies with univariate or multivariate analyses. DTCs, digestive tract cancers; OS, overall survival.

**Figure 4.**
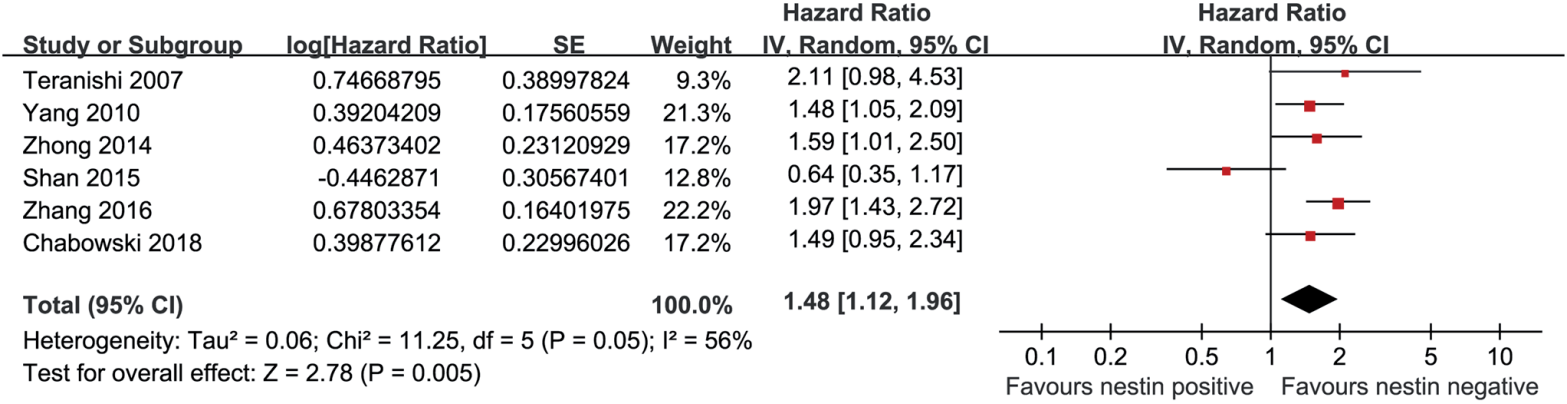
Forest plots for the meta-analysis of the association between nestin expression and DFS in patients with DTCs. DFS, disease-free survival; DTCs, digestive tract cancers.

Eleven studies^25,27-31,33,35-38^ reported the association between the level of nestin expression in cancer tissue and OS in patients with DTCs. Pooled results showed that higher expression of nestin (positive) in tumor was associated with worse OS (HR: 1.38, 95% CI: 1.11 to 1.72, *P* = .004, *I* ^2^ = 68%; Figure 2A) in patients with DTCs. Sensitivity analyses by excluding 1 dataset at a time showed consistent results (HR: 1.30 to 1.47, *P* all <.05). Subgroup analysis showed that higher nestin was associated with worse OS of GC (HR: 1.46, 95% CI: 1.23 to 1.74, *P* < .001, *I* ^2^ = 0%) and LC (HR: 2.05, 95% CI: 1.66 to 2.52, *P* < .001, *I* ^2^ = 0%), but not CRC (HR: 1.03, 95% CI: 0.69 to 1.53, *P* = .89, *I* ^2^ = 0%) or PC (HR: 0.96, 95% CI: 0.72 to 1.28, *P* = .80, *I* ^2^ = 26%; *P* for subgroup difference < .001; Figure 2B). These findings may also suggest that the location of the digestive cancers may be the source of heterogeneity of the meta-analysis (*I* ^2^ for overall meta-analysis = 68%, but became 0% in 3 subgroups and 26% in another subgroup according to the location of the cancer). Further subgroup analysis showed a consistent association between nestin expression and poor OS in Asians (HR: 1.46, 95% CI: 1.09 to 1.94, *P* = .01, *I* ^2^ = 76%) and non-Asians (HR: 1.21, 95% CI: 0.90 to 1.62, *P* = .20, *I* ^2^ = 24%; *P* for subgroup difference = .38; Figure 3A), and in univariate (HR: 1.27, 95% CI: 0.94 to 1.71, *P* = .11, *I* ^2^ = 62%) and multivariate studies (HR: 1.50, 95% CI: 1.11 to 2.03, *P* = .009, *I* ^2^ = 66%; *P* for subgroup difference = .44; Figure 3B). Besides, pooled results of 6 studies^26,28,32,34,35,37^ showed that higher expression of nestin (positive) in tumor was associated with worse DFS in patients with DTCs (HR: 1.48, 95% CI: 1.12 to 1.96, *P* = .005, *I* ^2^ = 56%; Figure 4). A sensitivity analysis excluding 1 study at a time did not significantly alter the results (HR: 1.37 to 1.68, *P* all < .05).

### Publication Bias

**Figure 5.**
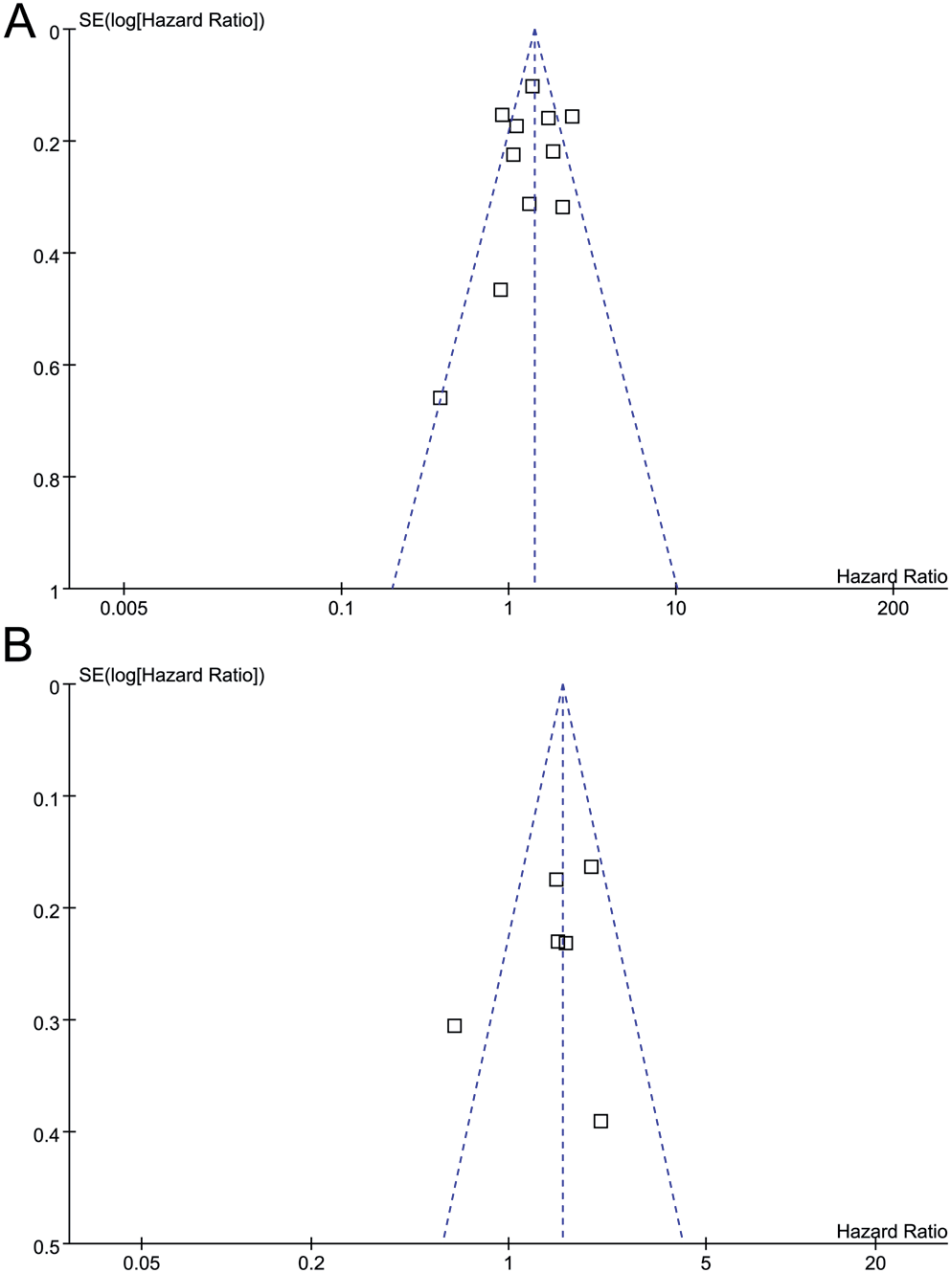
Funnel plots for the publication bias of the meta-analysis of the association between nestin expression and survival in patients with DTCs; (A) OS and (B) DFS. DFS, disease-free survival; DTCs, digestive tract cancers; OS, overall survival.

In Figure 5A and 5B, we show funnel plots illustrating the association between nestin expression and OS and DFS. As per visual inspection, the plots were symmetrical, suggesting a low risk of publication bias. As a result of Egger’s regression tests, publication biases were also considered to be low (*P* = .411 and .132, respectively).

## DISCUSSION

Based on the meta-analysis of fourteen cohort studies, it was found that higher levels of nestin in cancer tissue are associated with poorer survival, such as OS and DFS, in patients with DTCs. According to a sensitivity analysis, neither of the included studies contributed significantly to the results of the meta-analyses. Further subgroup analyses showed that the relationship between higher tumor expression of nestin and poor OS was consistent in Asian and non-Asian studies, and in univariate and multivariate studies. Besides, the subgroup analysis also suggested that differences in cancer type may significantly affect the association between nestin expression and OS. Specifically, higher tumor expression was associated with poor OS in patients with GC and LC, but not in patients with CRC or hepatic cancer. Taken together, these results suggested that higher nestin in cancer tissue is associated with poor survival of DTCs, particularly for patients with gastric and LCs. Since tumor expression of nestin could be easily evaluated by histologic analysis, such as IHC, incorporating nestin expression into the cancer histologic analysis may be of significance for the prediction of prognosis in patients with DTCs, such as GC and LC.

The study summarizes for the first time the association between tumor expression of nestin and patient survival outcomes in patients with DTC. Overall, the results of our meta-analysis are consistent with previous findings in other cancers such as breast cancer, NSCLC, ovarian cancer, and glioma, and collectively, these studies have found that higher nestin expression in cancer is associated with poor survival.^21-24,45^ Several mechanisms have been proposed underlying the possible association between nestin and progression of cancers.^46^ Previous experimental studies showed that nestin, as a member of intermediate filament proteins, is actively involved in the process of cancer development and metastasis via multiple mechanisms, including sustaining proliferative signaling pathways in cancer cells, such as the phosphoinositide 3-kinase/Akt pathway,^47^ evading tumor growth suppressors such as p53,^31^ promoting resistance of cancer cell to death signaling pathways,^48^ maintaining replicative immortality of CSCs,^17^ and inducing tumor angiogenesis.^49^ A better understanding of the molecular pathway underlying the association between higher nestin levels and poor survival in DTC patients is warranted in future research.

Our subgroup analyses showed that the relationship between higher tumor expression of nestin and poor survival in patients with DTCs was consistent in Asian and non-Asian studies, which may suggest that the ethnicity of the patients seemed to have no significant influence on the association. Moreover, both univariate and multivariate analyses concluded that higher nestin expression in DTCs is associated with poor survival, which may suggest that the above association may be independent of age and sex of the patients, and stage and grade of the cancer. Further confirmation of the robustness of the findings was provided by these results. Interestingly, the results of the subgroup also suggested that differences in tumor type may significantly affect the association between nestin and survival outcomes in patients with DTCs, which may be a potential source of heterogeneity among the included studies. We found that higher tumor expression was associated with poor OS in patients with GC and LC, but not in patients with CRC or hepatic cancer. These results may reflect the fact that DTCs are a group of heterogeneous cancers, and nestin may play different roles in different DTCs. The potential mechanisms underlying the possible different role of nestin in different DTCs, to the best of our knowledge, have not been evaluated in previous studies. To validate our findings in the future, large-scale prospective studies are required, as are preclinical studies to determine how the potential different DTCs work.

The results of our meta-analysis should be interpreted with awareness of our study’s limitations. First, all the included studies were retrospective, putting the results at risk of selection and recall biases. Therefore, larger multicenter prospective studies based on homogeneous populations are warranted in the future. In addition, the criteria for defining higher tumor expression of nestin or positivity of nestin were varying among the included studies, and a uniform definition has not been developed, which may also contribute to the heterogeneity among the included studies. Also, this is rather a meta-analysis of study-level rather than individual-level data. Accordingly, possible influences of patient characteristics on the outcomes were unable to be determined based on our study, such as age, sex, comorbidities, and anticancer treatments applied. Finally, some of the included studies were based on univariate analysis. Variables confounding the association between tumor expression of nestin and survival of DTCs may exist.

In conclusion, According to this meta-analysis, higher nestin expression in cancer tissue predicts poor survival in patients with DTCs, particularly in those with GC and LC patients. Although future large-scale prospective studies should be conducted to verify our findings, incorporating nestin expression into the cancer histologic analysis may be of significance for the prediction of prognosis in some patients with DTCs.

### Acknowledgments:

The authors are indebted to the authors of the primary studies.

## Figures and Tables

**Table 1.** Characteristics of the Included Studies

Study	Country	Design	Cancer Types	Sample Size	Number of Patients With Positive Nestin	Mean Age (Years)	Male (%)	Methods for Nestin Measuring	Cutoff	Follow-Up Duration (Months)	Outcome Reported	Variables Adjusted
Kim et al^25^	Korea	R	GC	61	30	62.5	75.4	IHC	Median	60	OS	Age, sex, depth of invasion, lymph node metastasis, tumor stage, and tumor size
Teranishi et al^26^	Japan	R	CRC	101	50	65.5	62.4	IHC	Median	33	PFS	None
Kawamoto et al^27^	Japan	R	PC	60	20	64.6	58.3	IHC	>10% of tumor cells	24	OS	None
Yang et al^28^	China	R	LC	314	157	50	84.7	IHC	Median	82	PFS and OS	Age, sex, tumor size, tumor stage, vascular invasion, and adjuvant therapy
Lenz et al^29^	Czech	R	PC	117	38	60.9	49.6	IHC	>10% of tumor cells	20	OS	None
Kim et al^30^	Korea	R	PC	42	15	64.5	66.7	IHC	>50% of tumor cells	67	OS	Age, sex, tumor stage, and histologic grade
Zhong et al^32^	China	R	EC	93	32	61	77.4	IHC	Mean OD > 0.09	60	PFS	Age, sex, tumor stage, histologic grade, and lymph nodes metastasis
Tschaharganeh et al^31^	Germany	R	LC	118	26	NR	90.7	IHC	>50% of tumor cells	84	OS	None
Liu et al^33^	China	R	GC	125	24	62.1	77.6	IHC	>50% of tumor cells	39	OS	None
Shan et al^34^	China	R	AC	102	28	65	60	IHC	>50% of tumor cells	60	PFS	Age, sex, and status of resection margin
Zhang et al^35^	China	R	LC	220	114	60	49.5	IHC	>10% of tumor cells	39	PFS and OS	Age, sex, tumor stage, histologic grade, and adjuvant therapy
Miao et al^36^	China	R	GC	383	169	NR	72.1	IHC	>20% of tumor cells	62	OS	Age, sex, tumor stage, histologic grade, and distant metastasis
Chabowski et al^37^	Poland	R	CRC	125	57	69.8	NR	IHC	Median	49	PFS and OS	None
Tampakis et al^38^	Switzerland	R	CRC	592	124	63.9	44.7	IHC	Regression analysis determined cutoff	60	OS	Age, sex, tumor stage, histologic grade, vascular invasion, and invasive margin

AC, ampullary cancer; CRC, colorectal cancer; EC, esophagus cancer; GC, gastric cancer; IHC, immunohistochemistry; LC, liver cancer; NR, not reported; OD, optical density; OS, overall survival; PC, pancreatic cancer; PFS, progression-free survival; R, retrospective.

**Table 2.** Details of Study Quality Evaluation Via the Newcastle–Ottawa Scale

Study	Representativeness of the Exposed Cohort	Selection of the Nonexposed Cohort	Ascertainment of Exposure	Outcome Not Present at Baseline	Control for Age and Sex	Control for Other Confounding Factors	Assessment of Outcome	Enough Long Follow-Up Duration	Adequacy of Follow-Up of Cohorts	Total
Kim et al^25^	0	1	1	1	1	1	1	1	1	8
Teranishi et al^26^	0	1	1	1	0	0	1	1	1	6
Kawamoto et al^27^	0	1	1	1	0	0	1	1	1	6
Yang et al^28^	0	1	1	1	1	1	1	1	1	8
Lenz et al^29^	0	1	1	1	0	0	1	1	1	6
Kim et al^30^	0	1	1	1	1	1	1	1	1	8
Zhong et al^32^	0	1	1	1	1	1	1	1	1	8
Liu et al^33^	0	1	1	1	0	0	1	1	1	6
Shan et al^34^	0	1	1	1	1	0	1	1	1	7
Zhang et al^35^	0	1	1	1	1	1	1	1	1	8
Miao et al^36^	0	1	1	1	1	1	1	1	1	8
Chabowski et al^37^	0	1	1	1	0	0	1	1	1	6
Tampakis et al^38^	0	1	1	1	1	1	1	1	1	8
